# Impact of Age on the Cancer-Specific Survival of Patients with Localized Renal Cell Carcinoma: Martingale Residual and Competing Risks Analysis

**DOI:** 10.1371/journal.pone.0048489

**Published:** 2012-10-30

**Authors:** Muyan Cai, Jinhuan Wei, Zhiling Zhang, Hongwei Zhao, Yunqiao Qiu, Yong Fang, Zhenli Gao, Jiazheng Cao, Wei Chen, Fangjian Zhou, Dan Xie, Junhang Luo

**Affiliations:** 1 Department of Urology, First Affiliated Hospital, Sun Yat-Sen University, Guangzhou, China; 2 State Key Laboratory of Oncology in South China, Cancer Center, Sun Yat-Sen University, Guangzhou, China; 3 Department of Urology, Cancer Center, Sun Yat-Sen University, Guangzhou, China; 4 Department of Urology, Affiliated Yantai Yuhuangding Hospital, Qingdao University Medical College, Yantai, China; 5 Department of Urology, First Affiliated Hospital, GuangZhou University of Chinese Medicine, Guangzhou, China; Chancellor College, University of Malawi, Malawi

## Abstract

**Background:**

Age at diagnosis has been shown to be an independent prognostic factor of localized renal cell carcinoma (RCC) in several studies. We used contemporary statistical methods to reevaluate the effect of age on the cancer-specific survival (CSS) of localized RCC.

**Methods and Findings:**

1,147 patients with localized RCC who underwent radical nephrectomy between 1993 and 2009 were identified in our four institutions. The association between age and CSS was estimated, and the potential threshold was identified by a univariate Cox model and by martingale residual analysis. Competing risks regression was used to identify the independent impact of age on CSS. The median age was 52 years (range, 19–84 years). The median follow-up was 61 months (range, 6–144 months) for survivors. A steep increasing smoothed martingale residual plot indicated an adverse prognostic effect of age on CSS. The age cut-off of 45 years was most predictive of CSS on univariate Cox analysis and martingale residual analysis (*p* = 0.005). Age ≤45 years was independently associated with a higher CSS rate in the multivariate Cox regression model (HR = 1.59, 95% CI = 1.05–2.40, *p* = 0.027) as well as in competing risks regression (HR = 3.60, 95% CI = 1.93–6.71, *p* = 0.001).

**Conclusions:**

Increasing age was associated with a higher incidence of cancer-specific mortality of localized RCC. Age dichotomized at 45 years would maximize the predictive value of age on CSS, and independently predict the CSS of patients with localized RCC.

## Introduction

Age at diagnosis has been demonstrated to be is a prognostic factor for the cancer-specific survival (CSS) of some kinds of human cancers, including renal cell carcinoma (RCC) [1]–[15]. These findings help oncologist to select more appropriate therapy strategy for different age group of cancer patients[16]–[20]. The previous studies always only considered cancer-specific mortality and neglected other cause mortality when age at diagnosis was demonstrated to be an independent prognostic factor for CSS, but other cause mortality rates may vary according to age, and other cause mortality reduces the pool of individuals at risk of cancer-specific mortality, these may lead to the overestimate of the impact of age on CSS. Recently, competing risks regression model was recommended to eliminate this limitation [21], [22].

Secondly, the selection of age cut-off point was arbitrary when the effect of age on the prognosis of cancer patients had been estimated in the previous studies. If statistical analysis was used to select the optimal age cutpoint which could best separate patients at a high and a low risk of cancer-specific mortality, the results would be more valuable for therapy selection and patient counseling. Herein, martingale residual analysis was used to select the optimal age cutpoint that would that would maximize the predictive value of age on the CSS of localized RCC, and competing risks regression was used to identify the independent impact of age on CSS by adjusting other cause mortality.

## Materials and Methods

### Patients

By using the departmental surgical database of our four institutions (First Affiliated Hospital and Cancer Center of Sun Yat-Sen University, Yantai Yuhuangding Hospital, and First Affiliated Hospital of GuangZhou University of Chinese Medicine), we identified 1,147 patients who had treated with radical nephrectomy for unilateral, sporadic localized RCC (T1–T2, N0, and M0) between 1993 and 2009. The ethics committees at Sun Yat-Sen University, Yantai Yuhuangding Hospital, and GuangZhou University of Chinese Medicine approved of the study and waived the need for consent. Data collected from each patient included age at diagnosis, gender, BMI (body mass index), ECOG PS (Eastern Cooperative Oncology Group performance status), tumor side, TNM stage, Fuhrman grade, histological subtype, and survival time. Tumors were classified in accordance with the 2002 TNM staging system. The grading system used in the study was based on the Fuhrman four-grade. Histological subtypes were stratified in accordance with the 2002 AJCC/UICC classification, and only tumors of clear-cell, chromophobe, and papillary histology were included. We classified cause of death as either RCC-related or as other-cause-related. Overall survival time was determined from the date of surgery to the date of death from any cause or last follow-up. CSS time was determined from the date of surgery to the date of death from RCC or last follow-up.

### Statistical Analysis

To identify a potential threshold between the younger and the older patients, a range of possible age cut-off values were estimated and tested in a univariate Cox proportional hazard regression model. In order to gain more insight into the dependence of CSS on age, a multiple Cox proportional hazard model was used [23]. Firstly, a Cox regression model excluding age as a factor was fitted. Then, functional shape was checked in the Cox regression model for the covariate by means of a smoothed martingale residual plot. A cut-off value should be considered if a steep increase or decrease of the resulting smoothing line crossed the null line of the x axis at the respective age.

The survival rates were calculated by the Kaplan–Meier method, and differences were determined by log-rank test. The prognostic significance of certain factors was assessed by the Cox regression model. The effect of other-cause mortality cannot be accounted for in Cox regression model. This may in turn result in the overestimate of the effect of cancer-specific mortality. The use of competing risks regression (Fine and Gray’s regression) can remove this limitation [24]. Competing risks regression was used to test the significance of cancer-specific mortality predictors after accounting for other-cause mortality.

Probability values of *p*<0.05 were considered significant. R version 2.11.1 (R Foundation for Statistical Computing, Vienna, Austria) was used for all calculations pertaining to Fine and Gray’s regression. The SAS version 9.1.3 (SAS Institute, Cary, NC, USA) was used for all other calculations.

## Results

### Clinical Outcome

**Figure 1:**
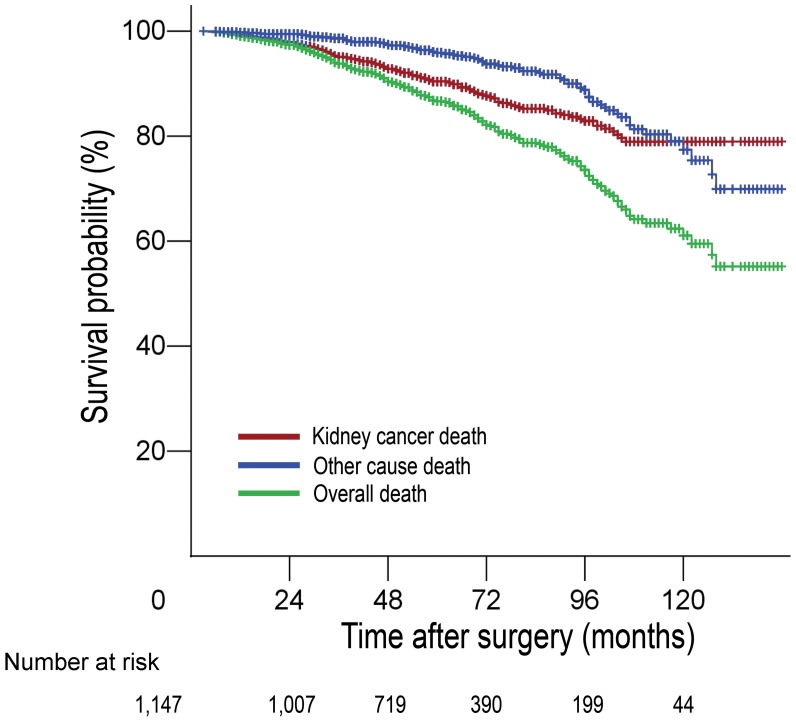
Predicted probability of cancer-specific survival, non-kidney cancer specific survival, and overall survival.

Patient demographic data are presented in Table 1. The mean age was 51.3 years (range, 19–84 years; median, 52 years). The median follow-up was 61 months (range, 6–144 months) for survivors. Of the patients, 124 of them died of RCC during follow-up, and 78 died of other causes. The 3-, 5-, and 7-year CSS rates were 95.1%, 90.4% and 85.2%, the 3-, 5-, and 7-year non-kidney-cancer survival rates were 98.7%, 96.3% and 92.3%, the 3-, 5-, and 7-year overall survival rates were 93.8%, 86.7% and 77.5%, respectively (Figure 1).

**Table 1:** Demographic and clinical data.

Characteristics	Results
Age, median (range), yr	52(19–84)
Gender, No. (%)	
Male	754(65.7%)
Female	393(34.3%)
Tumor side, No. (%)	
Left	591(51.5%)
Right	556(48.5%)
Histological subtype, No. (%)	
Clear	927(80.8%)
Papillary	161(14.0%)
Chromophobe	59(5.2%)
Fuhrman grade, No. (%)	
G1	383(33.4%)
G2	508(44.3%)
G3/4	256(22.3%)
Tumor stage, No. (%)	
pT1a	369(32.2%)
pT1b	465(40.5%)
pT2	313(27.3%)
BMI, No. (%)	
<25 kg/m^2^	720(62.8%)
≥25 kg/m^2^	427(37.2%)
ECOG PS, No. (%)	
0	903(78.7%)
≥1	244(21.3%)

### The Optimal Age Cutpoint Selected by Univariate Cox Regression

To identify the optimal age cut-off point that would maximize the predictive value of age on CSS, the patients were then divided into two groups at each age cut-off, from 35 years to 70 years, at five-year intervals. Univariate Cox regression demonstrated that the age cut-off of 45 years led to the highest significant difference in CSS between the respectively defined subgroups (HR = 1.78, 95% CI = 1.18–2.67, *p* = 0.005; Table 2).

**Table 2:** Univariate Cox proportional hazard regression model of different age cutoffs in patients with local RCC treated with radical nephrectomy.

Cutoff Point (years)	Categories	HR	95% CI	*p* value
**35**	≤35(n = 121) *vs* > 35(n = 1026)	2.07	0.96–4.43	0.062
**40**	≤40(n = 258) *vs* > 40(n = 887)	1.87	1.14–3.09	0.013
**45**	≤45(n = 408) *vs* > 45(n = 739)	1.78	1.18–2.67	0.005
**50**	≤50(n = 565) *vs* > 50(n = 582)	1.60	1.11–2.29	0.011
**55**	≤55(n = 711) *vs* > 55(n = 436)	1.49	1.05–2.13	0.025
**60**	≤60(n = 851) *vs* > 60(n = 296)	1.39	0.95–2.02	0.083
**65**	≤65(n = 961) *vs* > 65(n = 186)	1.40	0.91–2.16	0.123
**70**	≤70 (n = 1057) *vs* >70(n = 90)	1.46	0.82–2.60	0.194

### The Optimal Age Cutpoint Selected by Martingale Residual

**Figure 2:**
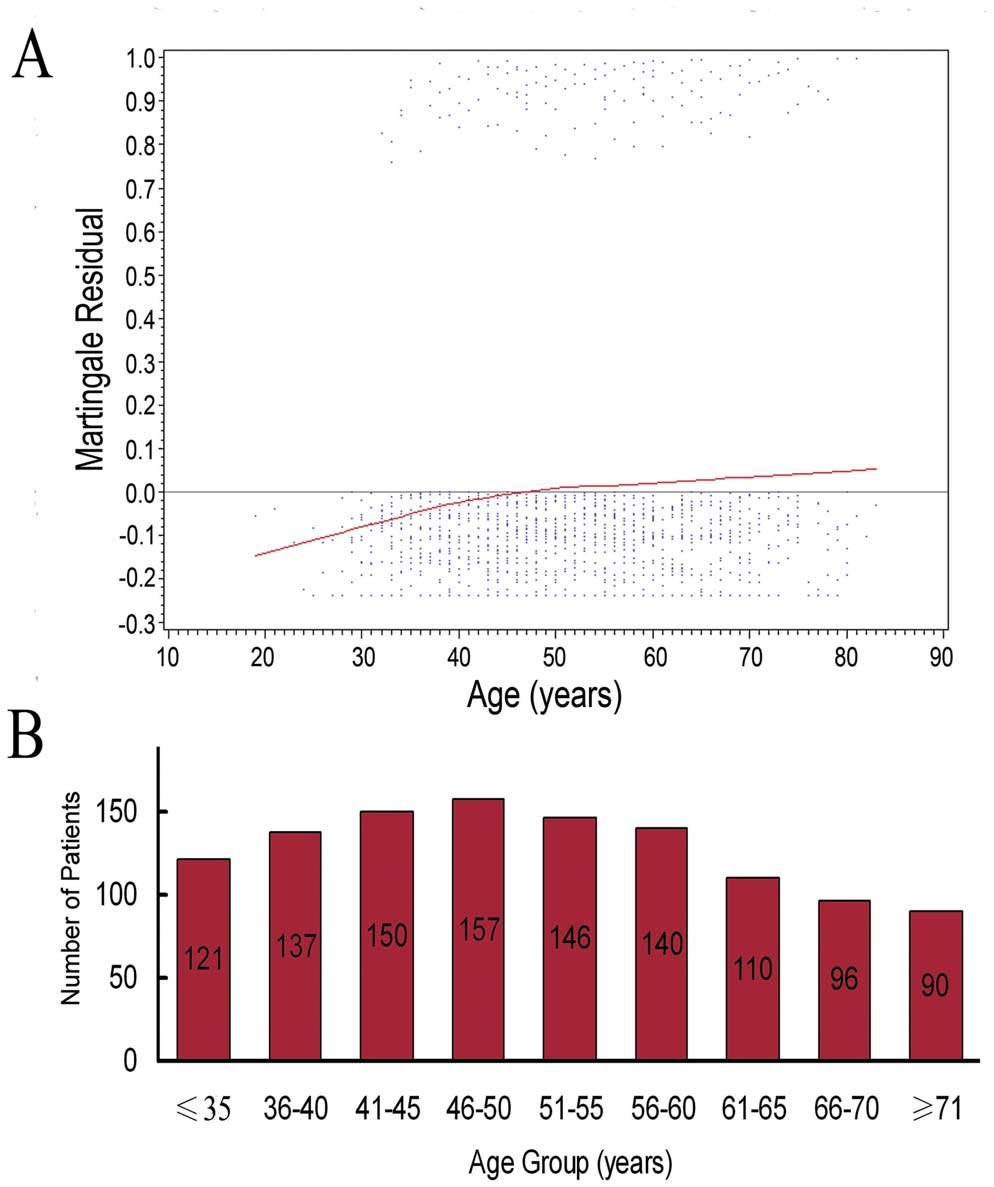
Martingale residual analysis. (A) Scatterplot of age *vs* martingale residual of Cox proportional hazard model. The smoothed curves crossed the null line at 46 years. (B) Age distribution of 1,147 patients with localized RCC.

The multivariate Cox proportional hazard model was used to further confirm the optimal age cut-off point. A steep increasing smoothed martingale residual plot indicated an adverse prognostic effect of age on CSS. The martingale residual curve crossed the null line at 46 years of age with martingale residual (Figure 2). Therefore, in multiple analysis, 45 years of age was also demonstrated to be the optimal age cut-off point that would maximize the predictive value of age on the CSS.

### Survival Analysis in the Cox Regression Model

We defined age ≤45 years old and >45 years old as the younger group and the older group, respectively. The association between CSS and age (HR = 1.59, 95% CI = 1.05–2.40, *p* = 0.027), Fuhrman grade (HR = 1.38, 95% CI = 1.08–1.78, *p* = 0.011), stage (HR = 1.42, 95% CI = 1.10–1.82, *p* = 0.006), and ECOG PS (HR = 1.63, 95% CI = 1.11–2.41, *p* = 0.013) were statistically significant in univariate and multivariate Cox regression analysis (Table 3).

**Table 3:** Univariable and multivariate analysis with Cox regression model for risk factors predictive of CSS.

Characteristics	Univariate Analysis		Multivariate Analysis	
	HR(95% CI)	*p* value	HR(95% CI)	*p* value
Gender (Male *vs* Female)	0.81 (0.55–1.19)	0.284	0.81(0.55–1.20)	0.300
Tumor stage (pT1a *vs* pT1b *vs* pT2)	1.76(1.39–2.23)	**<0.001**	1.42(1.10–1.82)	**0.006**
Age(≤45 *vs* >45 years old)	1.78(1.18–2.67)	**0.005**	1.59(1.05–2.40)	**0.027**
Histological subtype (Clear *vs* Papillary *vs* Chromophobe)	0.58(0.38–0.87)	**0.008**	0.69(0.46–1.04)	0.076
Fuhrman grade (G1 *vs* G2 *vs* G3/4)	1.64(1.28–2.08)	**<0.001**	1.38(1.08–1.78)	**0.011**
Tumor side (Left *vs* Right)	1.18(0.83–1.67)	0.366	1.19(0.83–1.70)	0.339
BMI (<25 *vs* ≥25)	0.67(0.46–0.98)	**0.041**	0.76(0.51–1.13)	0.170
ECOG PS (0 *vs* ≥1)	2.26(1.56–3.26)	**<0.001**	1.63(1.11–2.41)	**0.013**

### Survival Analysis Adjusted by Competing Risks Regression

**Figure 3:**
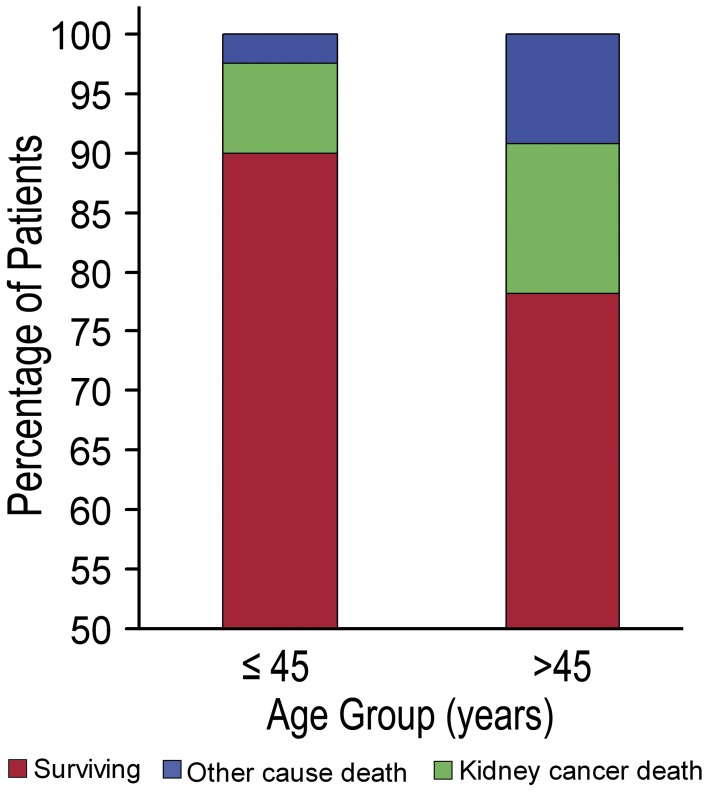
Distribution of health status, comprising survival probability and probability of death by RCC-cause death, and other-cause death.

**Figure 4:**
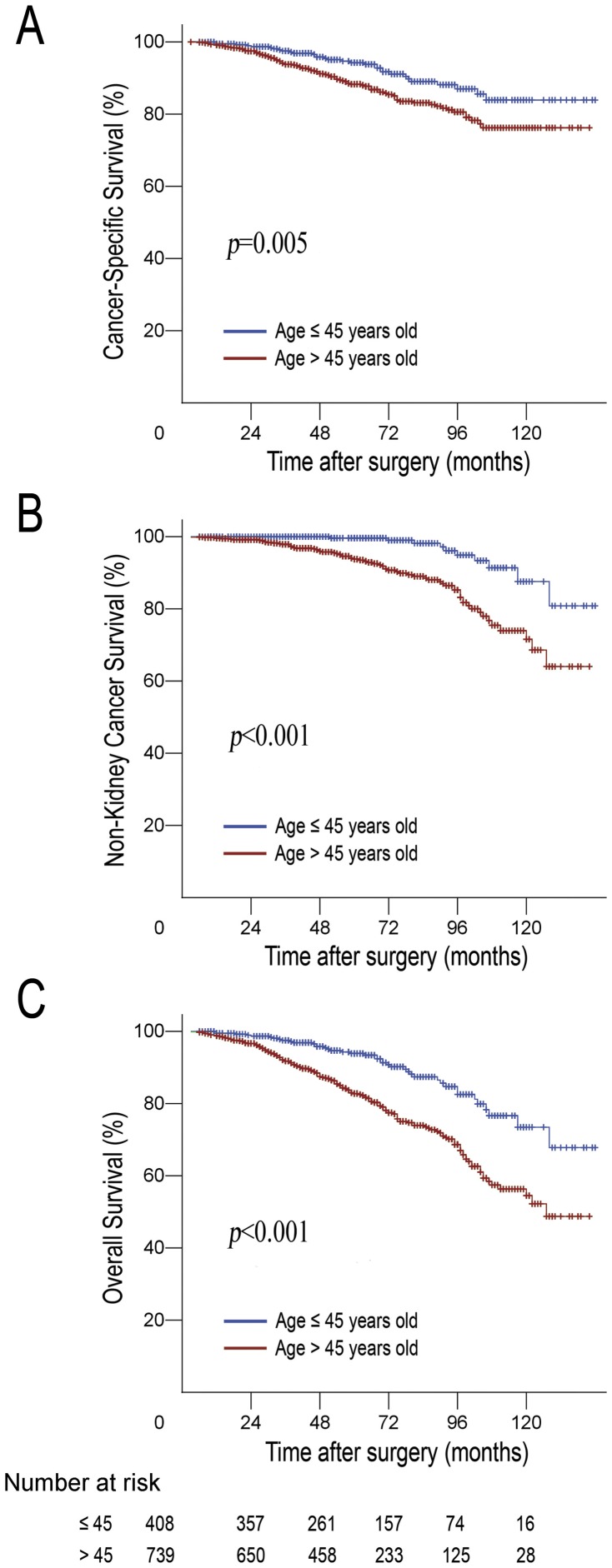
Predicted probability of (A) cancer-specific survival, (B) non-kidney cancer specific, and (C) overall survival by age shown using cumulative incidence function.

Of all patient deaths, 38.6% (78/202) were directly attributable to non-kidney cancer causes. Figure 3 presents the distribution of health status of the younger group and the older group, and the younger group patients had a significantly lower cancer-specific mortality, other-cause mortality and overall mortality (Figure 4). We complemented our analyses with competing risks regression, which addressed the significance of the combined multivariate contribution of all risk factors to cancer-specific mortality, after accounting for other-cause mortality. On multivariate competing risks regression analysis, an age of under 45 years was found to be an independent prognostic factor for better CSS of localized RCC (HR = 3.60, 95% CI = 1.93–6.71, *p* = 0.001). Of the other variables that were significant in univariate analysis, stage (HR = 0.73, 95% CI = 0.55–0.97, *p* = 0.030) and ECOG PS (HR = 2.73, 95% CI = 1.75–4.27, *p* = 0.001) were shown to be independent prognostic factors. However, pathologic subtype, and Fuhrman grade were no longer regarded as being the predictors of CSS (Table 4).

**Table 4:** Univariable and multivariable competing risks regression models for prediction of cancer-specific survival after accounting for other-cause mortality.

Characteristics	Univariate Competing Risks Regression		Multivariate Competing Risks Regression	
	HR(95% CI)	*p* value	HR(95% CI)	*p* value
Gender (Male *vs* Female)	0.80(0.54–1.18)	0.280	1.22(0.76–1.98)	0.404
Tumor stage (pT1a *vs* pT1b *vs* pT2)	1.45(1.12–1.88)	**0.004**	0.73(0.55–0.97)	**0.030**
Age(≤45 *vs* >45 years old)	1.52(1.01–2.29)	**0.044**	3.60(1.93–6.71)	**0.001**
Histological subtype (Clear *vs* Papillary *vs* Chromophobe)	0.60(0.39–0.91)	**0.018**	1.03(0.70–1.51)	0.883
Fuhrman grade (G1 *vs* G2 *vs* G3/4)	1.36(1.06–1.76)	**0.015**	1.03(0.75–1.42)	0.832
Tumor side (Left *vs* Right)	1.23(0.86–1.77)	0.250	0.89(0.56–1.40)	0.617
BMI (<25 *vs* ≥25)	0.69(0.46–1.03)	0.073	1.06(0.67–1.67)	0.791
ECOG PS (0 *vs* ≥1)	1.63(1.10–2.42)	**0.014**	2.73(1.75–4.27)	**0.001**

## Discussion

Several previous studies have indicated that increased age was correlated with poor CSS for RCC [9], [10]. Traditionally, the prognosis factors which are used to predict the CSS of patients with RCC also include stage, nuclear grade, histological subtype, tumor size, and performance status [25]–[27]. When a continuous variable was demonstrated to be a prognosis factor, the selection of the optimal cut-off point could maximize its predictive value and to best separate the patients with high risk of cancer death from patients with low risk due to tumor progression. Similar to the way in which tumor size is a predictor of CSS in localized RCC, increased tumor size was associated with poor CSS for localized RCC. Previous studies demonstrated that the 7 cm cut-off point led to the highest significant difference prognosis of localized RCC. Therefore, the T_1_ tumors were defined as being the organ-confined tumors of 7 cm or less in size, and the T_2_ tumors as being those larger than 7 cm in the current TNM classification of RCC [28], [29].

Taccon et al [12] demonstrated that young patients, defined as those who were ≤40 years old, had better 5-year CSS rates than older patients (90.8% *vs* 78.3%; *p* = 0.005). An age under 40 years old was an independent prognostic factor for the CSS of patients with RCC (*p* = 0.047). Taccon selected 40 years of age as the age cut-off point. This was based on previous studies concerning early-onset breast and colon cancers at or before 40 years of age, which suggested that there were differences in the natural history of these cancers for young patients. Jung et al [13] divided 619 patients with RCC into two groups according to age at diagnosis, ≤55 or >55 years of age. The younger group had a greater 5-year CSS rate than the older group (88.9% *vs* 76.3%, *p*<0.001), and Jung selected this threshold as a prognostic cut-off point because the median patient age was 55 years old. In two other similar studies, a young patient was defined as one being under 50 years old, CSS rates were also significantly higher for younger patients compared to the older patients with RCC [14], [15].

Martingale residual analysis is always used to identify the optimal cut-off point of a continuous variable to best separate patients at a high and a low risk of disease progression. Soares et al [30]estimated the effect of age on six-month survival of 862 patients who were critically ill with cancer, and martingale residual analysis was used to divide the population into young (<60 years) and elderly (≥60 years) groups with the largest survival difference. The prognostic significance of maximum tumor diameter was assessed in young patients who had good-prognosis diffuse large-B-cell lymphoma treated with CHOP-like chemotherapy plus rituximab. Martingale residual plots showed an adverse prognostic effect of maximum tumor diameter on event-free and overall survival, and a cut-off point of 10 cm separated two populations with the largest event-free survival difference [31]. Herein, martingale residual analysis was used to determine the optimal age cut-off point. A steep increasing smoothed martingale residual plot indicated an adverse prognostic effect of age on CSS. We found no cut-off point other than that of 45 years to be more suitable for distinguishing between the younger and the older patients with localized RCC that would maximize the predictive value of age on the CSS.

In the current study, over one-third of all patient deaths were directly attributable to non-kidney cancer causes. The adjustment for other-cause mortality is important because other-cause mortality reduces the pool of individuals at risk of cancer-specific mortality, and it leads to the overestimate of the importance of cancer-related deaths. The effect of other-cause mortality was neglected in Cox regression models; the use of competing risks regression models can eliminate this limitation [21], [22]. Several studies have shown that older men are more likely to be diagnosed as having high-risk prostate cancer and as having lower CSS [32], [33]. Recently, Bechis et al [22] investigated 11,790 men in the Cancer of CaPSURE database with complete risk, treatment, and follow-up information. Competing risks regression was used to identify whether a patient’s age at diagnosis is the independent predictor of his CSS. The study showed that age was a univariate predictor of both overall and prostate-cancer-specific mortality; unadjusted Kaplan–Meier survival curves showed decreasing survival rates among men with increasing age (*p*<0.01). However, on multivariate competing risks analysis, age was no longer a predictor of prostate-cancer-specific survival after controlling for treatment modality and disease risk. Therefore, an independent predictor of CSS in univariate and multivariate Cox regression models does not always have predictive value after accounting for other risks of death on competing risks analysis. Herein, multivariate competing risks regression analysis was used to further confirm the impact of age on CSS, and an age under 45 years old was found to be an independent prognostic factor for better CSS of localized RCC. Of the other variables that were significant in univariate analysis, stage and ECOG PS were shown to be independent prognostic factors. However, pathologic subtype and Fuhrman grade were no longer the predictors of CSS.

Herein, the study population comes from China, it is uncertain whether the conclusions in this study are accurate for the populations of other countries. However, the optimal cut-off point analysis and the competing risks regression analysis utilized in this study also have applicability to assess the impact of age on the prognosis of human cancers in other studies.

In summary, our study reconfirmed that the increasing age of patients with localized RCC was associated with a higher incidence of cancer-specific mortality. We demonstrated that 45 years old was the optimal age cut-off point that would maximize the predictive value of age on a patient’s CSS, and age dichotomized at 45 years old was an independent predictor of cancer-specific mortality in the multiple Cox regression model as well as in the competing risks regression. The optimal cut-off point analysis utilized in this study also has applicability to assess the impact of age on CSS of other cancers.
